# Supplementary material for: Resting-State EEG Alterations of Practice-Related Spectral Activity and Connectivity Patterns in Depression
Source: Biomedicines. 2024 Sep 10;12(9):2054. doi: 10.3390/biomedicines12092054 (PMC11428598; doi:10.3390/biomedicines12092054)
Supplement: Supplementary file 1 [file biomedicines-12-02054-s001.zip › biomedicines-3150621-supplementary.pdf]

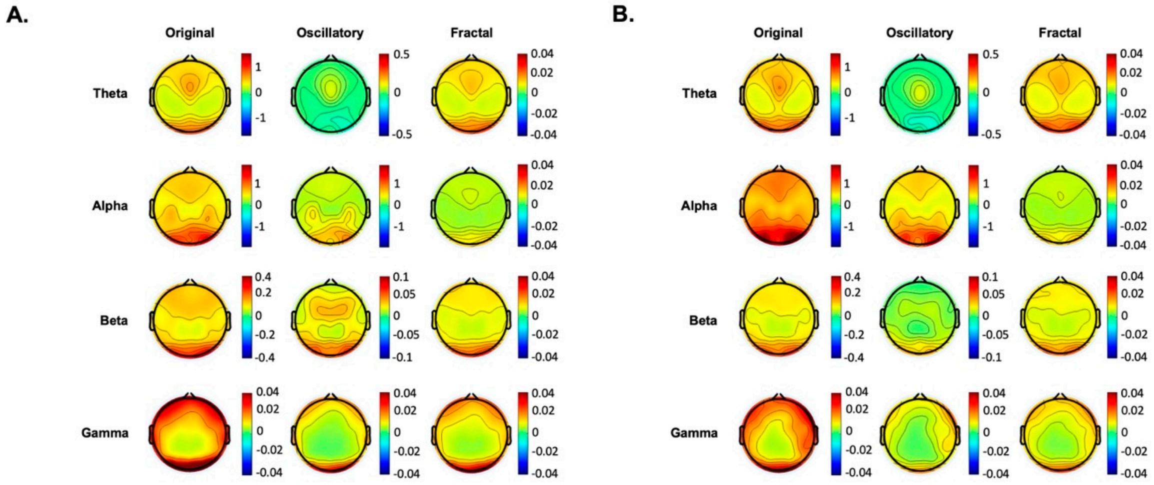

Figure S1 Topographic maps of the resting state EEG of the hBDI (A.) and CLT (B.) groups before the task, at the baseline. The three columns represent: the original (first column), oscillatory (second column), and fractal (third column) components for each frequency band.

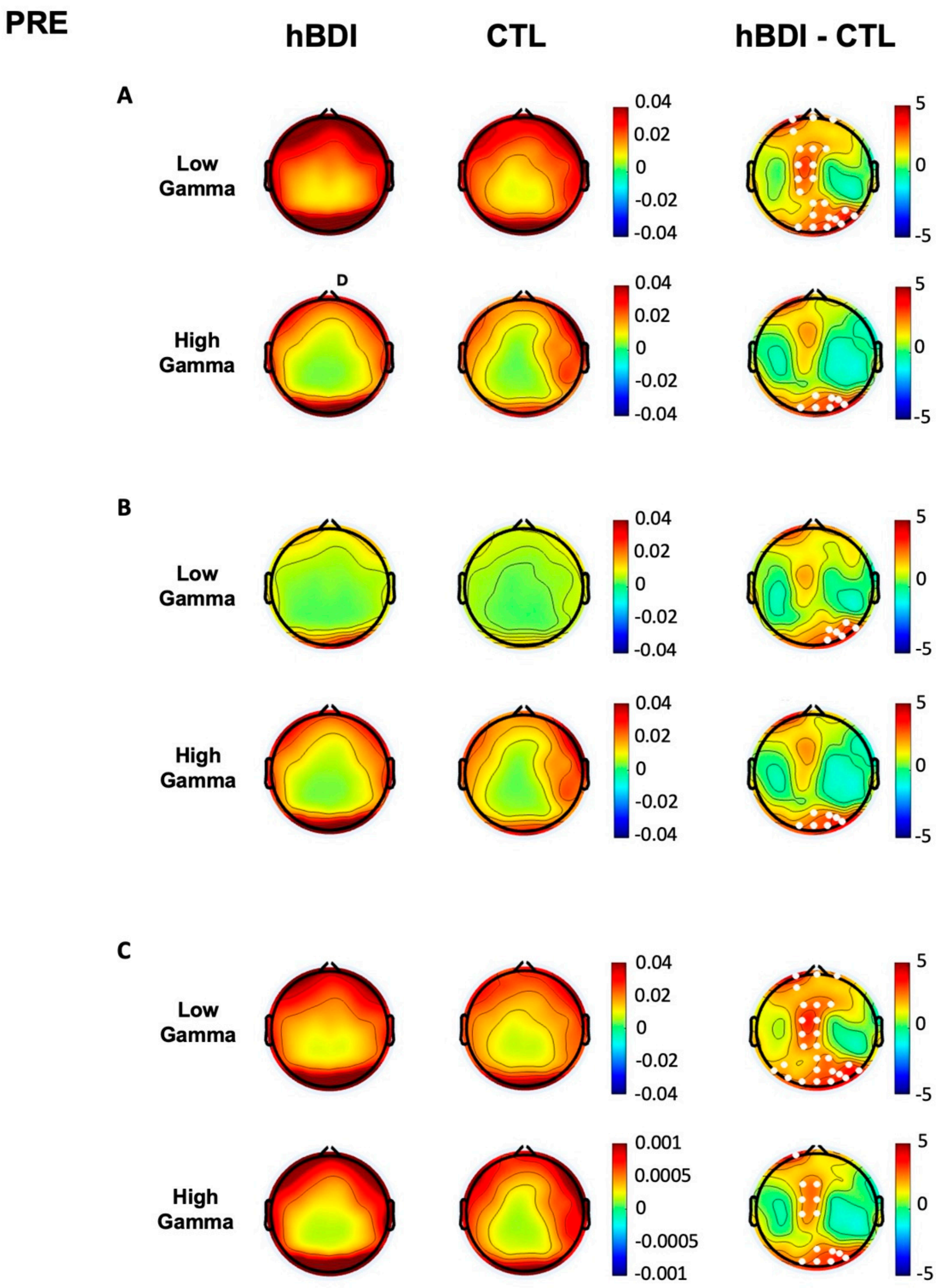

Figure S2 Topographic maps of high and low gamma frequency in the baseline pre-task recordings. Topographic maps of low gamma (from 30-58 Hz) and high gamma (from 62-90 Hz) activity in the hBDI (first column) and CTL (second column) groups before the task at baseline, for the original (A.), oscillatory (B.), and fractal (C.) components. The third column shows T-maps for group comparisons (hBDI vs. CTL). Significant group differences identified after cluster correction with multiple comparisons are denoted by white dots on electrode locations.

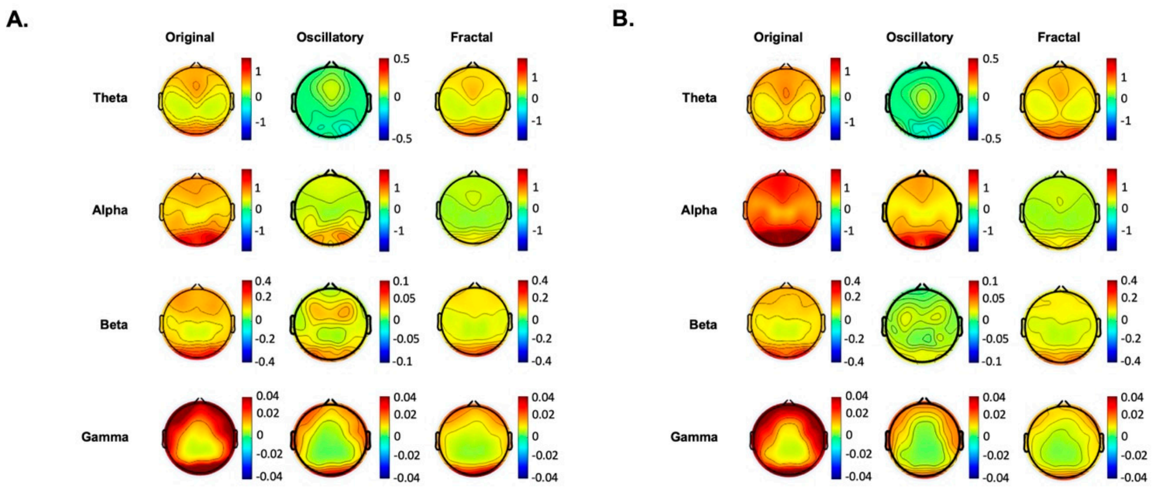

Figure S3 Topographic maps of the resting state EEG of the hBDI (A.) and CLT (B.) groups after the task. The three columns represent: the original (first column), oscillatory (second column), and fractal (third column) components for each frequency band.

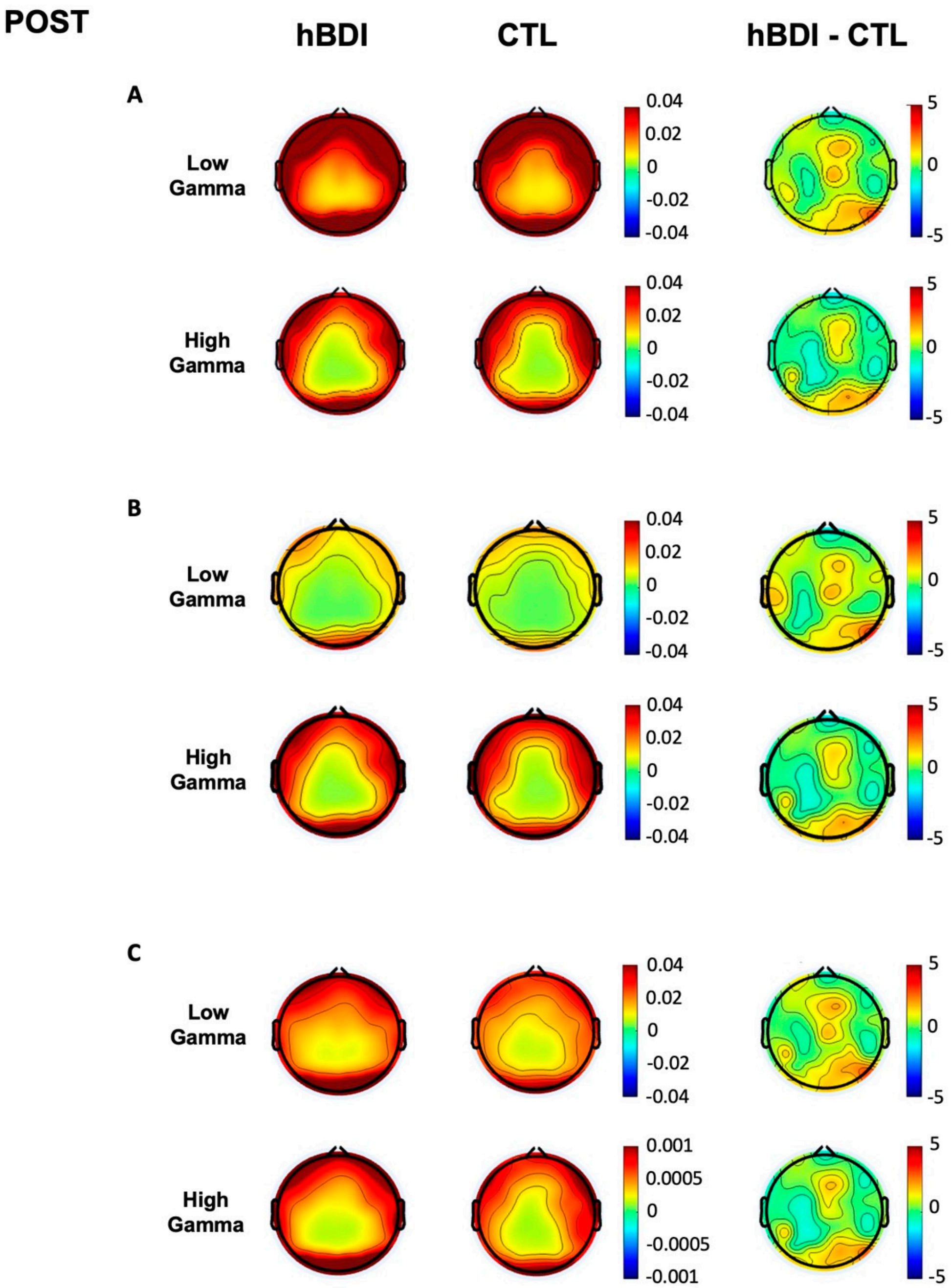

Figure S4 Topographic maps of high and low gamma frequency in the post-task recordings. Topographic maps of low gamma (from 30-58 Hz) and high gamma (from 62-90 Hz) activity in the hBDI (first column) and CTL (second column) groups after the task for the original (A.), oscillatory (B.), and fractal (C.) components. The third column shows T-maps for group comparisons (hBDI vs. CTL). Significant group differences identified after cluster correction with multiple comparisons are denoted by white dots on electrode locations.
